# “Responses to the Chilean law of food labeling and advertising: exploring knowledge, perceptions and behaviors of mothers of young children”

**DOI:** 10.1186/s12966-019-0781-x

**Published:** 2019-02-13

**Authors:** Teresa Correa, Camila Fierro, Marcela Reyes, Francesca R. Dillman Carpentier, Lindsey Smith Taillie, Camila Corvalan

**Affiliations:** 1 Faculty of Communication, Diego Portales University, Vergara, 240 Santiago, Chile; 2 Institute of Nutrition and Food Technology, University of Chile, El Líbano, 5524 Santiago, Chile; 3 School of Media and Journalism, University of North Carolina, Chapel Hill, NC 27599 USA; 4 Department of Nutrition, Gillings School of Global Public Health, University of North Carolina, Chapel Hill, NC 27599-7461 USA; 5 Carolina Population Center, University of North Carolina, Chapel Hill, NC 27516 USA

**Keywords:** Food Labeling, Front-of-package (FOP) labeling, Food Marketing, Food Regulation, Focus Groups, Chile, Understanding of the Regulation, Perceptions of the Regulation, Behaviors associated with the Regulation

## Abstract

**Background:**

In line with calls for action from international health organizations, Chile implemented in June 2016 a set of regulations to tackle the obesity epidemic. The new regulation includes the mandatory use of front-of-package warning labels on packaged foods/beverages high in energy, sugars, saturated fats and sodium. Additionally, such foods cannot be sold nor offered in daycares/schools and cannot be promoted to children under 14yo. The law is targeted to children; thus, this study examined mothers’ understanding, perceptions, and behaviors associated with the regulation one year after its implementation, using a qualitative approach.

**Methods:**

Nine focus groups of mothers (7–10 people each) of children (2-14yo) were conducted in July 2017 in Santiago-Chile. They were stratified by socioeconomic status (SES) and children’s age. Macrocodes were developed by three researchers, combining an iterative process of deductive and inductive thematic analyses. Quotations representing each category were selected.

**Results:**

Mothers understood the new regulation as a policy to fight child obesity and were aware that products with more labels were less healthy than products with fewer labels. Attention and use of labels in the buying decision-making process ranged from participants who did not pay attention to others who relied on them as a quick shortcut (mostly from middle and upper-SES); many mothers indicated changing their purchase habits only when buying new products. Mothers declared that young children accepted school environment changes while teens/preteens resisted them more. Many mothers agreed that schools have become key promoters of food behavioral change. Mothers were less aware about the food marketing regulations. Mothers declared that they perceived that the regulation was changing the perceptions, attitudes and behaviors toward healthier eating patterns.

**Conclusion:**

After the first year of implementation, the regulation was well known by mothers of diverse SES and different children ages. The degree of use of warning labels was heterogeneous among participants, but most of them agreed that their children, particularly the youngest have positive attitudes toward the regulation and have become promoters of change in their families. Many mothers also expressed that they perceived an important shift toward healthier eating, which may lead to a change in eating social norms. This information contributes to better understand how regulatory actions may influence people’s consumer behaviors.

## Background

Obesity has become a worldwide epidemic and public health authorities have urged countries to implement policies that address the increasing obesity and related non-communicable diseases, particularly among children [1, 2]. As a result, several strategies have been discussed and attempted, including mandatory easy-to-understand nutrition labels (e.g., Ecuador has a traffic light system) [3], marketing restrictions of unhealthy foods in child-targeted media (e.g., United Kingdom) [4], taxes on sugar-sweetened beverages and non-essential energy-dense foods (e.g., Mexico) [5] and policies that target school-food environments (e.g., Canada and Brazil) [6].

In Chile, obesity and diet-related diseases reach epidemic proportions. One out of four school children (24.6% 6-7yo old) and a third of adult population (31.2% in >15yo) present obesity [7, 8], while high body mass index and diet-related risk factors are the main cause of premature death and disability in the country. As a response, a comprehensive food regulation was introduced in June 2016 which combined several initiatives (front-of package (FOP) labeling, marketing restrictions, and school regulations) to promote healthier food environments through a multifactorial, structural approach [9, 10]; children were the intended primary beneficiaries.

Given the paucity of policy interventions, there is scarce evidence on their impact, especially when regulations are installed in combination such as in the case of the Chilean Law of Food Labeling and Advertising. A key component of the success of the policies is based on the perceptions that people may have of the different actions and how regulations influence their attitudes and behaviors. In Chile, mothers are a key stakeholder of the new policy because they are primarily responsible for food purchase decisions and serve as gatekeepers for food availability in the household [11], thus, understanding their perceptions is particularly relevant. Therefore, the purpose of this study is to examine mothers’ understanding, perceptions, and behaviors associated with Chile’s food regulation using a qualitative approach. We explored how each of the three key areas of the law—FOP labeling, school regulations, and marketing restrictions—are related to family eating and food purchase decisions and how they interact with each other. It is especially important to understand whether the  implementation of a package of measures improves results observed with single actions [12]. Thus, our findings should inform future regulations in Chile as well as in other countries.

## Methods

### Study design

We conducted qualitative focus groups in Chile’s capital city, Santiago, where over one third of the country’s population live. Nine focus groups of 7–10 mothers of children aged 2–14 were conducted in July 2017, one year after the introduction of the regulation. Focus groups allowed a deeper comprehension on how mothers understood, received and experienced the new policy as well as observing how they discussed about it. In focus groups, participants can interact, explore each other’s arguments and express topics that they deem important [13, 14]. The 9 focus groups allowed a 3 × 3 stratification according to socioeconomic status (lower, middle, upper) and children’s age (2–6; 7–10; 11–14). This stratification was based on previous research [15] and intended to obtain a diversity of views and experiences based on mothers’ SES background and their children’s development stage.

### Chilean law of food labeling and advertising

Details of the law have been described elsewhere [10, 11]. Briefly, the Chilean law of food labeling and advertising applies multiple marketing and sales restrictions to foods and beverages with high levels of energy, saturated fats, sodium and sugars (HEFSS hereafter). Thresholds to be considered high in critical nutrients have become more restrictive according to three phases of implementation of the law (see the thresholds for June 2016, June 2018 and June 2019 in Table 1).

**Table 1:** Critical Nutrients Thresholds of the Staggered Law Implementation

Solids	2016	2018	2019
Energy [kcal/100 g]	350	300	275
Sodium [mg/100 g]	800	500	400
Total Sugars [g/100 g]	22.5	15	10
Saturated fats [g/100 g]	6	5	4
Liquids	2016	2018	2019
Energy [kcal/100 ml]	100	80	70
Sodium [mg/100 ml]	100	100	100
Total Sugars [g/100 ml]	6	5	5
Saturated fats [g/100 ml]	3	3	3

Due to the law implementation, HEFSS products must include a front-of-package label (FOP, a black stop sign) that announces the high critical nutrient, for example, “high in sugar” or “high in sodium,” such that a product could have four labels if all four critical nutrients exceed the regulation’s thresholds. In addition, HEFSS products cannot be sold, distributed or promoted in daycares or schools, nor can these products be advertised in child-targeted media on the radio, television, cinema and internet. HEFSS marketing also cannot include strategies that appeal to youth up to 14yo, such as the presence of children, characters, celebrities, athletes, toys, or school references [10].

### Participants and recruitment

Mothers (*n* = 84) were recruited from 20 out of 35 districts of Santiago. A focus group recruiting company was hired to get access to mothers from different neighborhoods and socioeconomic profiles, to make sure they did not know each other. The filter questionnaire included participants’ age, marital status, district where they live, children’s age, type of daycare or school (public, semi-private, private), occupation, level of education, education and occupation of head of household, family income and possession of material goods (i.e., car, house, current bank account, housekeeping service, internet connection and type of healthcare system). People who had worked in the marketing and food-related industries (e.g., supermarkets, restaurants, retail companies) were excluded from the study.

Socioeconomic status (SES) was identified by the following variables: family income, possession of material goods, type of school attended by their children and district of residence. Lower-SES included mothers with monthly family incomes of US$750 or less, who did not own a house or a car and did not have household internet connection and were registered in the public healthcare system. Their children attended public schools and lived in districts with a high proportion of poverty, according to the Chilean National Socioeconomic Characterization Survey (CASEN) (e.g., El Bosque, Conchalí, Puente Alto, Cerro Navia) [16]. Middle-SES included mothers with monthly family income that ranged US$750-US$3800, owned a car (but not necessarily a house) and had household internet connection. They were registered in the private healthcare system, their children attended either semi-private or private schools and lived in districts with lower levels of poverty (e.g., Ñuñoa, Peñalolén, Huechuraba, and Santiago Centro). Finally, upper-SES included mothers with monthly family incomes over US$3800, owned at least one car, a house and had household Internet connection. They were registered in the private healthcare system, their children attended private schools and lived in districts with very low proportion of poverty (e.g., La Reina, Las Condes) (see Table 2 for a description of the socioeconomic profiles of mothers who participated in the study).

**Table 2:** Participants’ Sociodemographic Profile

		*Lower-SES* *(n = 29)*	*Middle-SES* *(n = 28)*	*Upper-SES* *(n = 27)*
Marital status	Married	58.6%	46.4%	62.9%
	Single	27.5%	35.7%	11.1%
	Divorced/Separated	13.7%	14.2%	25.9%
Mother’s education	Elementary education	0%	0%	0%
	Incomplete high school	27.5%	0%	0%
	High school	62.1%	0%	0%
	Higher education	10.3%	100%	100%
Attendance of children’s schooling system	Public schools	100%	3.6%	0%
	Voucher schools	0%	18%	0%
	Private schools	0%	67%	100%
Average household income	≤ US$750	100%	0%	0%
	US $750–1000	0%	7.1%	0%
	US $1000–1500	0%	53.6%	0%
	US $1500–3800	0%	39.3%	14.8%
	≥ US $3800	0%	0%	85.2%
Material household goods	Household Internet connection	0%	100%	100%
	Owns one car	0%	100%	100%
	Owns a house	0%	17.9%	100%
	Housekeeping service	0%	7.1%	85.1%

### Procedures

Focus groups questions guiding the discussion were elaborated by an interdisciplinary group of scholars from epidemiology, nutrition, public health and communication who have been evaluating the regulation from a variety of angles. Thirty questions covered the evolution of eating routines, buying decision-making processes, opinions and behaviors regarding FOP labels, school regulation and marketing strategies; we also asked participants about some of the marketing techniques that food industry has used to counteract the message of the labels. Focus groups were led by one of the investigators and two research assistants in the communication department of Diego Portales University in Chile  and lasted 90 min on average. Participants' names were changed to ensure anonymity. IRB approval was obtained from INTA’s and University Diego Portales' Ethics Committee. Signed informed consents for each of the participants were obtained by the investigators facilitating the focus groups before starting the sessions.

### Transcription and analysis

Sessions were audio taped and then transcribed by two trained research assistants. By relying on a hybrid process that combined deductive and inductive thematic analyses [17], the first author, corresponding author and a trained research assistant developed macrocodes based on the research questions that tackled the different aspects of the law and previous literature [18]. Then, transcripts were subjected to an iterative process of careful reading and rereading [19] conducted by these three people independently, who revised the original template and developed a coding scheme based on the previous questions of the focus groups (i.e. deductive process) and the new themes that were generated from the transcripts (i.e. inductive process). Then, all the transcripts were assigned specific codes according to the main categories and subcategories and the quotations that best represented each category were selected, translated into English and revised by the team of three bilingual researchers.

## Results

The report of results is organized according to three key areas covered by the new law: FOP labeling, regulation in schools and marketing strategies. Within each section, we described the different themes that were generated based on the analyses. Also, because the analyses revealed that the different aspects of the regulation are closely interrelated and interact with each other, we included a fourth section that analyzes the cross-cutting effects.

### FOP labeling

Results showed that in all the focus groups mothers understood that the new regulation, including FOP labels, was a policy to fight the high levels of child obesity and related diseases in the country. For example, one mother of a 5yo girl (Gina) gave the following response: “*Because of the high levels of obesity, hypertension and diabetes”* and “*Some kids are very chubby. So (…) you can think that the alimentary habits need to be changed.*”

#### Awareness and uncovering

Everybody were aware that a product can be labeled with four signs maximum and that products with more labels are less healthy than products with fewer labels. Many mothers said they use the number of labels as guidance. For instance, Soledad, secretary at an educational institution with a 6yo boy, explained:

*“The good thing is that my son likes cookies a lot, so I tell him (in the supermarket) ‘look for the cookies that have the fewest labels and that one you can take. So, it is useful for me that he, by himself, realizes what is bad. He tells me, ‘Mom, look, this one has three (labels)” No, too much. Look for another that has one.*

The appearance of warning labels also “*uncovered*” many products, according to the mothers. Some admitted that they were surprised, saying that their “*eyes were opened*.” For instance, Dafne, homemaker (with 13yo and 23yo sons), who participated in the lower-SES group asserted: “*I didn’t think these things were so bad*.” Some said that they felt “*cheated*” by products they considered healthy such as breakfast cereals, cereal bars or yogurts. For instance, Constanza, homemaker from lower-SES (13yo son and 8yo daughter) explained that she used to buy a chocolate breakfast cereal but now she is changing it for an oat breakfast cereal. She also said: “(…) *regarding the cereal bars, I used to believe they were healthy and used to give them as a snack (to the kids), but they have too much sugar*.” Disappointment was even stronger in brands that were advertised and named as healthy and nutritious. For example, Dominique (5yo boy), used to buy muffins of the brand *Nutrabien (Goodnutrition)* as school snack:

*I associated that the brand Nutrabien (Goodnutrition) was very healthy, until those black labels came out. I realized that it had high levels of everything, and I felt very cheated (…) I really had no idea, I never paid attention. Now, I do pay attention.*

Despite that mothers were aware that the more labels, the unhealthier the product, they indicated that they do not understand well the principles that define when the packages have to carry labels. Regardless of participants’ SES, they said that they would like to have more information about the process of assigning labels to a product. For instance, one participant said: *“I would really like (…) more information (about FOP labels), because I’m not an expert in this topic” (*Anita, children 4yo and 10yo). *“It could be clarified (…) how much percentage of an ingredient a certain product should have” (*Gina, daughter of 5yo).

#### Gradational attention and use of labels

Analyses also revealed that there are gradations of attention and use of FOP labels in the buying decision-making process. While some mothers admitted they do not pay attention to them (e.g., *“I practically don’t see them,” “I don’t pay attention,” “I don’t use them”)*, others explained that they rely on them as a quick shortcut: “*I don’t read them like this (very closely) but when I see too many, I don’t buy it*.” (Delia, cashier, children of 6 m, 4yo and 13yo). Many said that they changed their purchase habits only when they buy new products. For instance, Patricia, dance instructor (mother of two children, 5yo and 8yo), explained: *“I tend to buy the same things as always. I only pay attention to these labels when I want to try out something new, I want to make sure that it doesn’t have a lot of sodium and sugar.”*

Other mothers, however, particularly from middle and upper-SES, asserted that they pay very close attention. Dominique (business administrator, 5yo son) said: *“The thing of labeling food products, I tell you… it did change my shopping decisions. Before, I used to buy thinking that everything was good and I didn’t read the nutritional table, now I do look at it.”* These changes were, many times, led by children. Mabel, homemaker from upper-SES (19yo daughter and 13yo son), explained:

*To be honest, I started to notice them [the labels] not by myself, but because my children, or their friends (…) now that this thing of the black labels started, start to read and be like ‘Mom, the yogurt has zero fats’ or… I don’t know how this is called… fat free.*

Despite the awareness and use of black labels, participants warned about the potential negative effects that might cause the omnipresence of the labels because many packages carry them. In a middle-SES focus group, the following dialogue was sustained: “*Sometimes [these labeling measures] I feel they’re invasive. The information can cause the reverse effect.*” (Claire, midwife, 4yo daughter). “*It (can cause) rejection*.” (Daisy, 6yo boy). “*Right, it’s rejection because in the end everything has labels…*” (Claire). “*It’s like that you don’t see the labels anymore*.” (Daisy).

### Regulation in schools

HEFSS foods cannot be sold at schools. Therefore, the on-site school kiosks –usually used to buy food during school breaks- have had to adjust what food they offer. Consuelo (veterinarian, two children of 4yo and 5yo) remembered that in her old school “*everything was fried. Now you go and it’s full of fruits, veggies, natural stuff*.” The analyses revealed that these changes have not been questioned by young children, however, they have been more challenging for teens and preteens, many of whom do not want to buy food at school. For instance, Maribel, a mother of a 13yo child, asserted:

*“My son didn’t agree when the school started to put restrictions on the things that couldn’t be sold, because he used to take cash to the school and would buy a hot dog and a soda (…) When they took everything away from the kiosk, [I’d ask] ‘what did you buy?’ ‘Nothing because everything was boring.’ ‘What was boring?’ ‘There were fajitas with veggies but there wasn’t ketchup and mayonnaise to put it in the fajita. They also sold fruits, and I didn’t like that.’ ‘So, what did you buy?’ ‘Nothing.’ So, he prefers to bring the money back home.”*

Other participants indicated that their children decided to change their diet. Paula (artisan) explained that her 13yo son “*used to buy fries with the money he took to the school. Then, I didn’t give him money anymore because he is tall and overweight, so he wouldn’t be able to buy fries and stuff like that. After that, the woman that sells food at my kid’s school started to sell fruits, sandwiches with avocado, with ham or cheese. Then my son started to take money back again, and he buys for himself a juice and a sandwich*.”

#### School as an agent of change

Despite the fact that the regulation at schools only established restrictions in terms of promotion and selling, in every focus group, mothers said that the school has become a key promoter of food behavioral change by explaining what is healthy or unhealthy. According to the mothers, teachers use the FOP labels as a shortcut by saying, for example, “don’t bring food with more than two labels.” Furthermore, they described that in elementary schools, it is no longer allowed to buy HEFSS food, and teachers are the ones that suggest what snacks are healthy enough to be acceptable. Some schools even organized a list with a day-by-day suggested healthy morning snack.

Some of these participants, particularly from lower-SES, explained that before this law, schools allowed and promoted special events (such as the last day of class) that involved the whole class in sharing snacks that were usually unhealthy (e.g., chips, sugar-sweetened beverages). But, within this past year, these gatherings and special events have no longer included unhealthy food because teachers do not allow these products in the classroom anymore. Instead, “*these gatherings with food for special events are now healthier at school*.” (Patricia, lower-SES focus group). “*They have fruit and sandwiches*” (Vania), “*olives*” (Patricia), “*carrot sticks*” (Vania).

Some participants agreed with this new policy, but others found it “*boring*” because the kids “*don’t eat, they leave everything on the table*” (Solange, 13yo daughter). Another mother explained that the family has been adjusting to the changes. For the shared-snack days and other parties hosted at the school, “*we may not bring fruit kebobs because they may not eat them, but we bring yogurt and [breakfast] cereal (…) or ham and cheese sandwiches*.”

#### Parents’ resistance vs. young children’s commitment

In lower-SES groups, a few participants expressed uneasiness about the new school food environment and rejected these changes. Some moms complained that their freedom to choose products for their children was diminished, some saying that they had to stop giving their children “*junk food*” for school snacks because those snacks could be taken away from their children at school. One participant acknowledged that now they were surrounded by a healthier environment, so she asserted that sometimes it is good to spoil children with “*junk food*”: *“Playground: healthy, home: healthy, TV: healthy. Sometimes it is good to spoil them. It is not like it’s going to happen every day. If almost everyone eats [junk food], why should we forbid them?”* (Vania, mother of a 5yo girl).

Despite the rejection of some mothers from lower-SES, their own children seem to be more committed with this law, especially children under 8yo, who started to ask for healthier snacks, replace juice and soda with water and consume more vegetables. This pattern was witnessed in every focus group, but it was more relevant and pervasive in the middle- and lower-SES. For instance, Gina (mother of a 5yo daughter) explained:

*Because of this new law, my daughter has been taught a lot about these black logos. ‘No mom, you can’t buy me that, my teacher won’t accept it because it has those labels’. And she requests me salads, she doesn’t accept snacks that have black labels. And because I have adapted to that as well, when we go grocery shopping, I see a product and I’m like… ‘No, she won’t accept that if I buy it to her’, so I have to search for a product that at most contains 2 logos. But three, there is no way.*

### Marketing strategies

#### Unnoticed strategies

Although mothers were aware that the law involved the FOP labels and restrictions in schools of unhealthy products, they were less aware that the law also regulated food marketing and advertising in the media or on product packages. Only a few participants had noticed that some of the front packaging on breakfast cereals no longer included cartoons or animals, such as the bunny from the Trix Cereal [10].

Following the mandatory FOP labels, a few companies have attempted to decrease the warning effects by including other labels on the same package. To offer an example of this marketing strategy, a package of cookies can include labels such as: Suggested portion = 3 cookies → 101 cal; 100 g. = 15 cookies → 3 logos ‘high in sugars,’ ‘high in saturated fats,’ ‘high in calories.’ The focus group participants had barely noticed these suggested additional labels. Some mothers thought that these suggestions were part of the regulation and were not aware that were a marketing response from the industry. Furthermore, some mothers stated that they did not understand what the label was suggesting. For instance,

*“I think it is not clear. People see so many labels (...) but they don’t understand that this happens (being high in critical nutrients) if you eat the whole package, but if you eat two or three (cookies), it does not affect you”* (Patricia, 5yo and 8 yo children).

In addition, the industry also used the reduction or lack of FOP labels as a marketing strategy, which elicited confusion and affected the credibility of FOP labels among a few participants. For instance, a dairy brand advertised that all its products were free of labels, including caramel and chocolate-based puddings. In addition, a chocolate breakfast cereal also reformulated its ingredients and was free of labels. Some participants explained that they were skeptical and suspicious when they noticed that some products had no labels because they had anticipated that these products would receive at least one label. Therefore, they wondered about reformulation and the nutritional composition that the industry was using to be qualified as a label-free product. Regarding this discussion, Paulette, psychologist, said: “*I really don’t believe this thing of black labels. I don’t even believe in light products. I don’t believe in that stuff because I think… ‘what are they putting [into the food]’ because now everything is processed, everything.”*

### Cross-cutting effects

Focus groups revealed that the different aspects of the law are closely interrelated and interact with each other. In the focus group discussion people integrated different areas of the law (i.e., FOP labels, regulation at school, marketing on TV) as a package of measures that fostered a change of attitudes and behaviors toward healthy eating. For instance, Adela (2yo daughter), said: “*I believe that there is a change of culture in a short time, a change that has been promoted by schools, (and) in television.”* In a similar vein, another participant asserted that this change toward healthier eating *“is promoted on TV, on the radio, and also informed at school. Now at school they won’t sell that much of junk food. It started to become massive.”*

Daycares and elementary schools are becoming more restrictive with the snacks that are allowed, and children, particularly younger children from lower-SES, have embraced this new healthier culture, discussing about the “black labels” at home and requesting their mothers to buy them healthier snacks. This is related to the appearance of FOP labels and campaigns promoted by the Ministry of Health in public and state-funded schools. As a result, many mothers admit that they have changed their children’s diet, especially school snacking. For instance, Carla, mother of a 9yo child, explained*: “My son eats at school. He, by his own, started to decide what he can eat and what not, this because of these black logos that are in the packaging.”*

Camelia, mother of a 13yo girl, clearly explained how the different aspects are intertwined:

*This topic about food diet and healthy snacks has been on the rise. First, we saw a flurry of information on TV, and then in schools. And let’s say that your children, because of all the information they receive at school, then they transmit it to you at home. Because of that, you have to change the option of what you give them as snack. For example, the thing that happens with cookies, now you give your kid cookies to take to school, and you’ll be like ‘what kind of mother am I.’ It appeared on the news and everywhere that the worst that you can give your children are cookies.*

Mothers admitted they felt a pressure from the school –and society in general—about their children’s healthy eating. Participants, particularly from middle and upper-SES, expressed that they felt “*guilty*” and “*bad mothers*” if they did not send healthy snacks to school:

*P1: When they [the children] got home with all these [information about healthy snacks] from school, I had to assume… all their classmates will take healthy snacks, so they cannot take chips.*

*I: How did you feel? Observed?*

*P1: No, you feel [being] bad mother.*

*P2: Yes, I feel being terrible mom when I see her eating chips. I let her do it sometimes because in a party I won’t say ‘don’t eat’ but I know it’s not good for her.*

## Discussion

In this qualitative study, we explored how Chilean mothers of young children understand and perceive the new regulation of food labeling and advertising. We found that mothers were aware that the more stop signs, the unhealthier the product and that many of them declare using FOP labels, particularly when purchasing new products. We also found that mothers perceive that school environments have become healthier as a result of the implementation of the law, although other aspects of the marketing restrictions are rarely perceived or noticed. Interestingly, the mothers’ discourses reflect that the effects of the different aspects of the law (FOP, school environments and marketing regulation) are all interrelated and operate in coordination to promote healthier behaviors. They also show that children, particularly young children from lower- and middle- SES, have become key disseminators of the messages underlying this regulatory effort.

FOP labels have been indicated as a key measure for the prevention of obesity [12, 20, 21]. Evidence suggests that the simpler the message, the higher the impact on consumer’s behavior [22]. Furthermore, research has also found that warning monochromatic FOP labels that flag products high in key critical nutrients improve consumers’ abilities to identify unhealthy food compared to Guideline Daily Amounts (GDA) and traffic-light systems [23, 24]. The results of this study are in line with this evidence. Chile has implemented a simple warning message (i.e., directive FOP) that aims to decrease the consumption of unhealthy foods. We found that mothers of different SES understand well the intended message (i.e., the more stop signs, the unhealthier the product), although one potential risk is that they understand that a product with one label corresponds to a healthy product. Mothers did not comprehend the underlying principles that explain in which cases a certain product should be labeled. Given that the process of purchase takes place in seconds, this is an intended outcome of a directive FOP like the one implemented in Chile [25, 26].

We also found that the implementation of the logo allowed “*uncovering*” some products. That is, it has helped in clarifying the lack of healthiness of some food products that had been traditionally advertised as healthy food. Oatmeal cookies, breakfast cereals, cereal bars, among other food categories have traditionally use healthiness as their marketing strategy and mothers were surprised when they found that the products had warning labels. In addition, the level of attention and use was gradational: mothers reported that the presence of warning labels influenced their purchase decision mostly when deciding about new food products. This result is in line with current FOP label research, which suggests that the impact on behaviors is less strong for products in which there is a consumer loyalty [27]. Finally, regarding FOP labels, mothers’ discourses also revealed that they sometimes felt that the pervasive presence of them was overwhelming and may not end up contributing to better decision making. Although it is not possible to rule out this possibility in the short term, future research could explore whether the omnipresence of warning labels may desensitize people in the long run.

Regulation at schools established restrictions in terms of promotion and selling of unhealthy foods and this was clearly perceived by mothers who participated in the focus groups. Many of them declared that these restrictions are “forcing” healthier children’s behavior because they do not have the option of buying unhealthy food products, particularly in the case of adolescents that bring money to the school; some mothers thought this was something desirable for shaping individual’s behaviors but other mothers, particularly those from low SES, declared that they found restrictions were unwarranted. From the mothers’ accounts, the analyses also revealed that the schools are going beyond the regulation because teachers are promoting that children should bring healthier snacks from home. Promoting healthier food environments at schools has also been identified as a key area of action in obesity prevention [28, 29] because there is evidence that shows how influential the food environment is, particularly among young children [30, 31]. This environment has a very important impact on their food routine behavior, considering that about one third of their total daily energy is consumed at educational establishments [32, 33]. This is why some governments have decided to take responsibility by becoming stricter with dietary routines within schools and banning HEFSS food from being sold inside the schools [6, 34].

In line with this evidence, mothers of young children declared that their children are strongly influenced by the teachers’ promotion of healthier diets, particularly with respect to school snacks. In fact, some of the mothers of young children felt that there was some kind of demonization of those bringing unhealthy snacks and, in some cases, they found that this healthy fashion was excessive. Among middle-SES mothers, there was a clear social pressure regarding healthiness of food to the point that they declared feeling guilty when giving unhealthy snacks to their kids. These discourses revealed both a change in eating patterns and a certain resistance to this new environment, which is expected according to research on behavioral change [35]. Overall, as a result of the healthier supply at schools and the role of teachers as health promoters, in every focus group, mothers assured that the school has become the main agent of food behavioral change, highlighting the key role of schools on obesity prevention [36].

In the case of marketing restrictions, the results showed that for many mothers the changes were unnoticed. The persuasion literature suggests that information is processed through two routes: the central route requires high cognitive elaboration and effort and the peripheral route –which requires lower involvement and thinking—also has an impact based on simple associations and cues [37]. Marketing strategies, such as child-targeted figures, likeable character or celebrity endorsements, are processed through peripheral routes, which means that the person does not invest much thinking about the object. Nonetheless, marketing has an impact on the consumer because strategies can make the product -in this case the food- attractive, eliciting a positive feelings through associations. Therefore, it is not surprising that the mothers of young children have not noticed the changes in marketing strategies, which does not necessarily imply that marketing restrictions have not had an impact on consumer’s behaviors.

Besides the marketing restrictions, the industry has responded with several advertising strategies that include competing FOP labels that suggest ideal portion sizes of consumption. Many of them had gone almost unnoticed by participants or cause some confusion. A few people thought that they were part of the regulation. In addition, messages about being a logo-free product or a logo-free brand elicited skepticism among mothers –they did not believe that some products that looked unhealthy were not subject to be labeled- and triggered a negative halo effect, in which a few participants extrapolated the skepticism toward a specific product and became suspicious about the global regulation [38].

Finally, the focus groups revealed cross-cutting effects, that is, the different aspects of the law are closely intertwined. Currently, schools not only restrict the selling of “labeled” products inside their facilities, but they have become key promoters of healthier food and snacks. The (lack of) warning labels are also present in the marketing strategies employed by the food industry, particularly on television. Thus, the discourses about the importance of healthier food environments are more pervasive. Young children have been receptive to these messages and changes in the discourses and behaviors and use the labels as a useful shortcut to categorize healthy vs. unhealthy food. As a result, they have become change agents in their families by forcing or persuading their mothers to change some food consumption habits. In some cases, there has been resistance, skepticism and criticisms about possible oversaturation, but the fact that people think and discuss about these issues mean that the discourses about health and food are pervading different sectors of society and may have long-term effects through changes in social norms.

A key strength of this study was the use of focus groups, which provided the opportunity to understand the complexities of how a new policy is received, discussed and experienced by one of the groups impacted by the regulation. Particularly, focus groups allowed observing how mothers of young children –a key stakeholder of the new policy—discussed and expressed their ideas about the perceived opportunities, strengths, challenges and shortcomings of the regulation and eventually understand more clearly their discourses, attitudes and self-reported behaviors after the law was implemented. Although the dynamics of focus groups may be led by a few dominant voices and participants may tend to acquiesce in front of the researcher, the investigator explicitly stated that “there were no right or wrong opinions or answers.” Then, with the help of a research assistant, purposefully asked questions that avoided social desirability and acquiescence response bias and encouraged the participation of all the members in each section of questions. To avoid group thinking, they used follow-up questions to clarify consistencies or inconsistencies with participant’s previous opinions. Another limitation is that the law itself could have increased social desirability bias and might have altered the answers mothers gave with regards to their food habits and beliefs in a social group. To avoid social desirability, future research could also use on participant observations in the households and schools. These results should be contrasted and complemented with quantitative research that examines family food habits. Finally, we recruited mothers because the evidence shows that they are the main food gatekeepers in the household [11]. In addition, most single-parent households are led by women and only 1,6% by men [39]. Although a very small percentage are led by men, future studies might include their perspective.

## Conclusions

After the first year of implementation, the focus groups revealed that the regulation was well known by mothers who belonged to lower, middle and upper SES and had children of different ages. The degree of use of warning labels was heterogeneous among participants, but most of them agreed that their children, particularly the youngest ones, had positive attitudes toward the regulation due to its high dissemination in schools and daycares. Many mothers also expressed that they perceived an important shift toward a healthier dietary pattern, which may lead to a change in eating social norms. We believe the present study contributes to better understanding how regulatory efforts can modify people’s behaviors and what are the potential pathways implicated in the positive and negative responses of consumers. This type of information results critical for understanding the impact of regulations as well as for guiding future adaptations or complementary actions.

## Abbreviations

FOP: Front-of-package
HEFSS: High energy, saturated fats, sodium and sugars
SES: Socioeconomic status
yo: Year old
